# A Dynamic and Pseudo-Homogeneous MBs-icELISA for the Early Detection of Aflatoxin B_1_ in Food and Feed

**DOI:** 10.3390/toxins15110660

**Published:** 2023-11-16

**Authors:** Lin Wei, Deyan Xu, Bei Yuan, Chengchen Pang, Haitao Xu, Kunying Nie, Qingqing Yang, Sibel A. Ozkan, Yanyan Zhang, Yemin Guo, Xia Sun

**Affiliations:** 1 School of Agricultural Engineering and Food Science, Shandong University of Technology, No. 266 Xincun West Road, Zibo 255049, China; weilin0127@163.com (L.W.); deyan1517@163.com (D.X.); yuanbei0605@163.com (B.Y.); pcc0319@163.com (C.P.); sdutxht0320@163.com (H.X.); kunying1015@163.com (K.N.); zyyan1104@163.com (Y.Z.); gym@sdut.edu.cn (Y.G.); sunxia2151@163.com (X.S.); 2 Shandong Provincial Engineering Research Center of Vegetable Safety and Quality Traceability, No. 266 Xincun West Road, Zibo 255049, China; 3 Zibo City Key Laboratory of Agricultural Product Safety Traceability, No. 266 Xincun West Road, Zibo 255049, China; 4 Department of Analytical Chemistry, Faculty of Pharmacy, Ankara University, 06560 Ankara, Türkiye; sibelaysil@gmail.com

**Keywords:** aflatoxin B_1_, monoclonal antibody, magnetic nanobeads, enzyme-linked immunosorbent assay

## Abstract

Aflatoxin B_1_ (AFB_1_) is one of the most toxic and harmful fungal toxins to humans and animals, and the fundamental way to prevent its entry into humans is to detect its presence in advance. In this paper, the monoclonal antibody mAbA2-2 was obtained via three-step sample amplification and multi-concentration standard detection using a subcloning method based on the limited dilution method with AFB_1_ as the target. A dynamic and pseucdo-homogeneous magnetic beads enzyme-linked immunosorbent assay (MBs-icELISA) was established using the prepared antibody as the recognition element and immunomagnetic beads as the antigen carrier. The MBs-icELISA showed good linear correlation in the concentration range of 0.004–10 ng/mL with R^2^ = 0.99396. The limit of detection (LOD) of the MBs-icELISA for AFB_1_ was 0.0013 ng/mL. This new ELISA strategy significantly shortened AFB_1_ detection time through improved sensitivity compared to the conventional ELISA method.

## 1. Introduction

Agricultural products are highly susceptible to fungal infections during processing, storage, and transportation, and toxins are highly harmful to humans [1,2]. The most common fungal toxins in agricultural products are aflatoxins (AFs), ochratoxins (OTA), vomitoxins (DON), zearalenone (ZEN), and T-2 toxins [3,4]. Among them, AFs are the most toxic and have strong carcinogenic, mutagenic, teratogenic and immunosuppressive properties [5]. More than 20 aflatoxin derivatives have been identified, four of which are produced under natural conditions, namely AFB_1_, AFB_2_, AFG_1_, and AFG_2_ [6,7]. AFB_1_ is the most prominent representative toxin in AFs and one of the genotoxic carcinogens [8]. It was classified as a Class I carcinogen by the World Health Organization Agency for Research on Cancer in 1993 [9,10,11]. In the molecular structure of AFB_1_, dihydrofuran is the basic toxin structure and oxanaphthone is the main structure causing carcinogenesis [12]. Early detection of aflatoxin contamination in food is one of the most fundamental ways to prevent aflatoxin from entering humans [13].

There are multiple quantitative methods for detection of AFs, the most authoritative being chromatographic (mass spectrometry–liquid chromatography, thin-layer chromatography) and biochemical methods [14,15,16]. However, these methods require extensive, specialized and expensive instruments, are cumbersome, and require preconcentration during the sample processing stage. With the development of biotechnology, biosensors using antibodies and aptamers as recognition elements are also rapidly developing [17,18]. Compared with aptamers, antibodies have superior specificity and affinity. Immunoassay techniques that focus on antigen-antibody-specific recognition, such as ELISA [19], chromatographic immunoassay [20,21,22], colorimetric immunoassay [23], electrochemiluminescence immunoassay [24], and fluorescence immunoassay [25], have been significantly investigated. Immunoassay techniques can be divided into heterogeneous and homogeneous immunoassays according to the state of matter [26]. Homogeneous immunoassays are performed in solution and require very few samples to be tested after a simple pretreatment, which can be applied to the detection of samples with more complex composition [27]. The most widely used immunoassay method is ELISA. Traditional ELISA is time consuming, requiring at least 5 h for a single assay, and a heterogeneous immunoassay confines the reaction surface to the bottom of the enzyme-labeling plate [28,29]. Zhang et al. [30] established an MBs-based direct competition ELISA based on the competition between free AFB_1_ and AFB_1_-CMO-HRP for MBs-mAbs binding sites by immobilizing mAb on magnetic beads (MBs) to improve the sensitivity of enzyme immunoassays. Li et al. [31] used magnetic nanochains instead of microplates as stationary phases to immobilize mAb while acting as stirring bars to facilitate liquid mixing and mass transfer, and AuNPs were used to coimmobilize HRP and detect antibodies. An ELISA with magnetic beads as the stationary phase kept the reaction system in a homogeneous state and improved the binding rate, which shortened the experimental time and saved reagents, as well as detection efficiency [32].

The quality of the antibody directly determines the sensitivity of the sensor, and the prerequisite for obtaining monoclonal antibodies is screening out high-quality positive hybridoma cells. Current positive hybridoma cell screening methods at the laboratory stage include the membrane immunoglobulin directed hybridoma screening and cloning (MIHS) method [33], cell surface fluorescence immunosorbent assay (CS-FIA) [34], and limited dilution method subcloning [35]. The membrane immunoglobulin directed hybridoma screening and cloning method (MIHS) and cell surface fluorescence immunosorbent assay (CS-FIA) either use flow cytometry for cell–cell separation or fluorescent materials for cell labeling, which are inevitably affected by the physical effects of flow cytometry and the chemical effects of fluorescent materials [36,37,38,39]. The traditional method of antibody preparation is to select cells in the wells of cell culture plates based on the amount of antibody in the supernatant of the cell culture medium, and to complete the purification and screening of positive hybridoma cells by selecting cells with good antibody quality from the wells for cloning through restriction dilution [40,41]. However, the traditional restriction dilution method has a long screening cycle, is costly, and high-quality positive hybridoma cells are easily covered and lost. The screening step can be accelerated by improving the medium required for cell growth, which is a growth environment and a source of nutrients. Zhang et al. [42] used the reported “novel two-step screening method” to screen AFB_1_ hybridoma cells. Yang et al. [43] used alternating liquid medium–semi-solid medium screening method to obtain capsaicin-like substance hybridoma cell lines. These studies established a screening method that accelerates the cell screening cycle by improving the cell culture medium approach based on the traditional cell screening method.

This study used the class distribution function vertex subcloning method combined with three-step sample amplification and multi-concentration standard assay screening to obtain AFB_1_ monoclonal antibodies for constructing a dynamic homogeneous immunomagnetic bead analysis method. In the cell detection after fusion, we found that the cell pore detection data distribution was similar to the distribution function, and the cell pore near the distribution function point is the better cell pore. Selecting these cells for sub-cloning, we defined this sub-cloning method as the class distribution function fixed-point sub-cloning method. Cellular pores at the apex of the class distribution function had the highest sensitivity to AFB_1_, and were selected for subcloning. Firstly, the cells were domesticated by reducing their concentration of AFB_1_. Secondly, the cells screened by a single standard were amplified with antibody samples after multiple subcloning and detected sequentially using multiple gradient concentration standards. Finally, the cells with reasonable specificity, affinity and stability of the secreted antibody were selected for antibody preparation. In this experiment, the MBs-icELISA was constructed using the prepared antibodies (mAbA2-2) as recognition primitives. The use of immunomagnetic beads expands the reaction vector from the bottom surface of a plate to the spherical surface of an infinite number of beads, significantly increasing the specific surface area of the vector, while the applied conditions keep the reaction system in a dynamic homogeneous process. Using immunomagnetic beads and additional conditions improves the binding rate and sensitivity of the assay.

## 2. Results

### 2.1. Screening and Characterization of Monoclonal Antibodies

#### 2.1.1. Effect of Immunization in Mice

**Figure 1:**
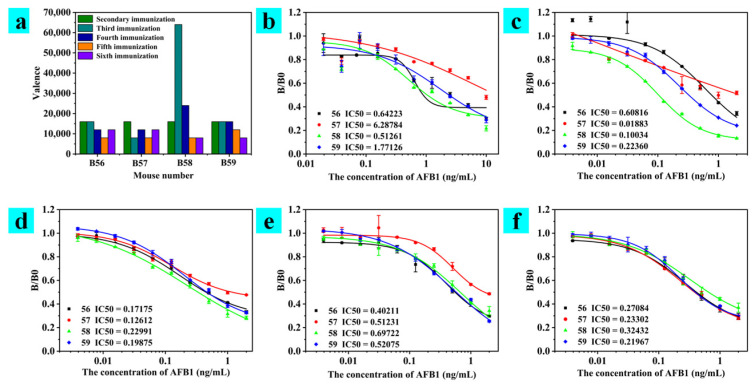
Serum detection data of mice after different times of immunization. (**a**) Determination of serum titers in mice with different immune times. (**b**–**f**) Specific detection data of ABF_1_ in serum from mice immunized from the second to the sixth time.

The serum indirect ELISA test data of mice after different immune time are shown in Figure 1. The B0 value was the absorbance value detected at 450 nm for the reaction solution without AFB_1_ for indirect ELISA detection, the B value was the absorbance value detected at 450 nm for the reaction solution with AFB_1_ for indirect ELISA detection, and the B/B0 value is the absorbance ratio with and without AFB_1_. As the number of immunizations increased, the mouse spleen cells became more mature. In the early stages of immunization, the immune system of mice produced IgM antibodies with a high splicing value but a short half-life. Therefore, in the early stages of immunization, the potency of the mouse serum assay was higher. Membrane-bound monomeric IgM (mIgM) antibodies were the hallmark of immature B cells. With repeated immunizations, the mouse immune system responded again by secreting IgG-type antibodies with a longer half-life, and the serum potency (Figure 1a) and specificity of the mice to the target object (Figure 1b–f) were also improved. After repeated immunizations, the spleen cells of mice matured to form large numbers of B cells. Immature B cells express only mIgM, while mature B cells may express both mIgM and mIgD, and were referred to as initial B cells. In the serum test after the last immunization, the titers of the mice were all above 16,000 (Figure 1a), among which the potency of B56 and B57 were 32,000, and of B59 and B57 were 16,000. Serum titers met the requirements of cell fusion. B56 and B57 mice had already reached 32,000 by the sixth immunization, but combined with the trend of previous serum potency changes, B56 mice were more in line with the trend of antibody potency changes. Their IC_50_ in the competition test was also pretty good. Their IC_50_ levels in the competition test were also positive, ranging from 0.2–0.35 ng/mL in the sera of all mice examined, as shown in Figure 1f. The inhibition curve of mouse B57 did not visually show a promising trend of inhibition in previous immunizations. The final ranking of the immunization effect of this mouse was B59, B56, B58, B57.

#### 2.1.2. Hybridoma Cell Subclones

**Figure 2:**
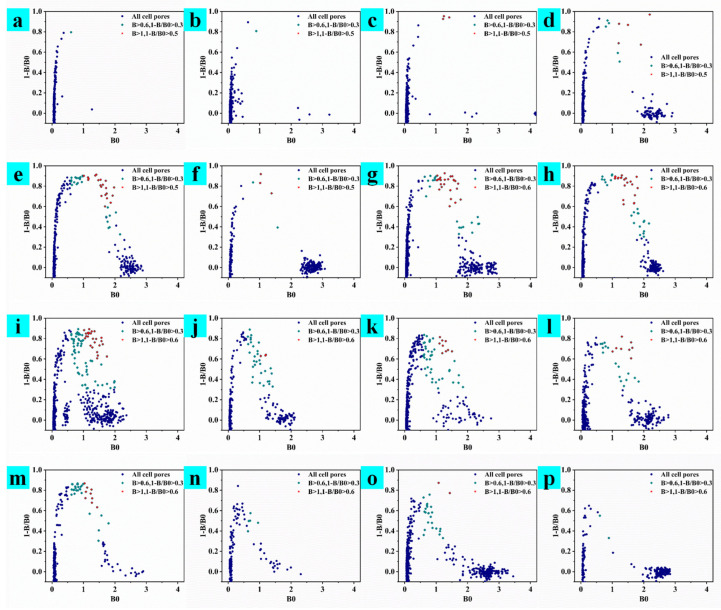
Subclonal supernatant detection data of the first mouse after fusion. (**a**–**c**) The first to third supernatant detection data of the primary cell plate. (**d**–**p**) Data from the 1st to 13th subclonal cell supernatant tests.

**Figure 3:**
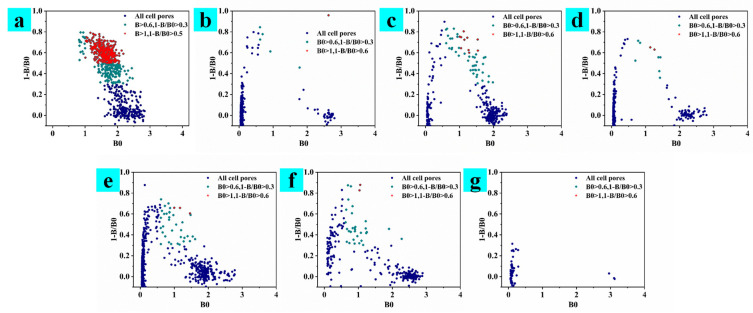
Subclonal supernatant detection data of mouse B59 after fusion. (**a**) Supernatant detection data of the primary cell plate. (**b**–**g**) Data from the 1st to 6th subclonal cell supernatant tests.

**Figure 4:**
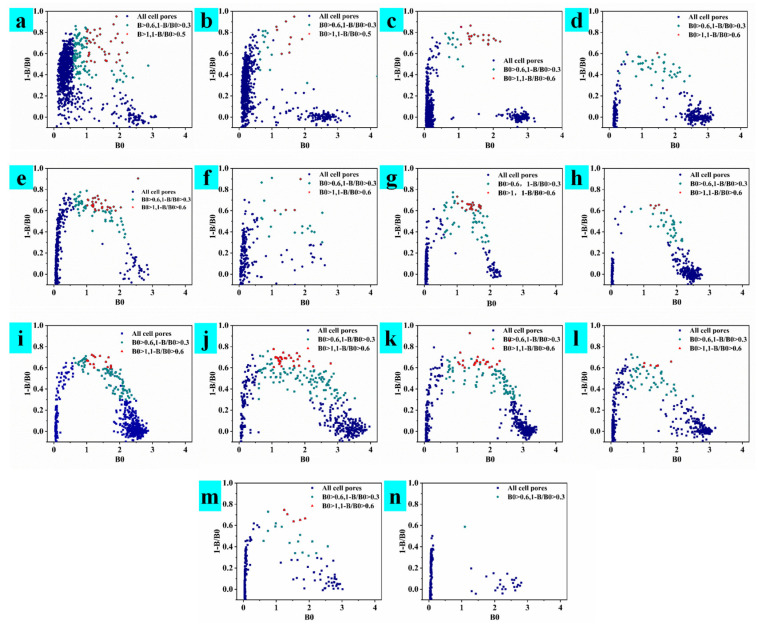
Subclonal supernatant detection data of mouse B56 after fusion. (**a**–**c**) The first to third supernatant detection data of the primary cell plate. (**d**–**n**) Data from the 1st to 13th subclonal cell supernatant tests.

In this experiment, three mice were used for cell fusion three times, and three different cell supernatant detection strategies are, respectively adopted; the results are shown in Figure 2, Figure 3 and Figure 4. A mouse that was well immunized in the laboratory was taken for cell fusion, named AFB_1_-0519, and the supernatant was detected when the cells in the subclonal cell hole grew to 1/6 of the cell hole, as shown in Figure 2. After the end of immunization, B59 and B56 mice were selected for cell fusion. If there were multiple cell lines in the cell wells, the undesirable cells grew faster than the desired cells, which overwhelmed the desired cells. Therefore, in the process for screening B59 mouse fused cells, the supernatant assay is performed when the cells grew to a population of approximately 1000, as shown in Figure 3. However, as the cells grew, the environment in the cell culture medium varied between the early and late stages of cell growth. Therefore, during the B56 cell screening process, the cell supernatant was assayed after two days of culture, following a single cell culture medium change when the cell population reached approximately 500, as shown in Figure 4.

**Figure 5:**
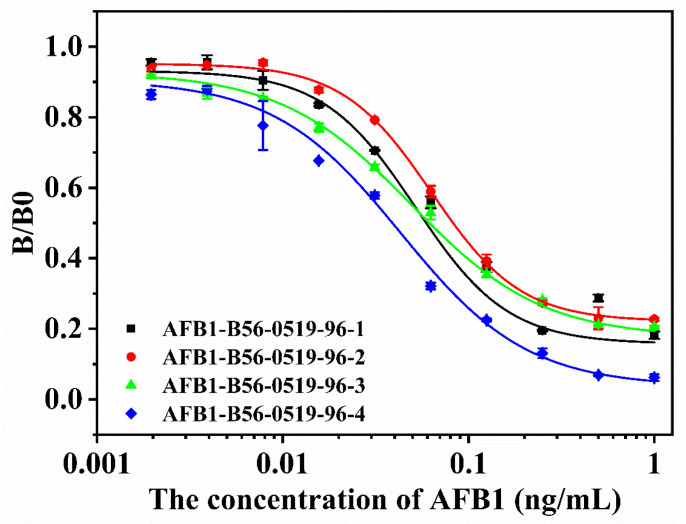
Supernatant assay of the tenth subclonal collection of 96-well cells.

Observing the cell assay plots of the three fusions (Figure 2, Figure 3 and Figure 4), we observed the number of cell pores with B0 > 0.6 and 1-B/B0 > 0.3, gradually increasing from AFB_1_-0519 to B59 to B56 as a whole. Observing the data plots, it could be seen that the cell pore distribution in the supernatant assay was somewhat similar to the distribution function; furthermore, the affinity and specificity of the cell pores near their vertices were higher. As shown in Figure 2, AFB_1_-0519 fusion cells were tested three times for supernatant assay and thirteen times for subcloning of the original cell plate. The positive rate of the subcloned plate gradually increased with the subcloning until the sixth subclonal assay reached the highest, after which the positive rate fluctuated up and down. As shown in Figure 3, the first positive rate of the fused protoplasts of B59 mice was 99.99%. However, the positive rate dropped sharply as the subcloning proceeded and kept fluctuating up and down, causing the loss of the positive cell line after the sixth subcloning. As shown in Figure 4, the original cell plate of B56 mice underwent three supernatant assay tests and thirteen subcloning assays after cell fusion. With the subcloning, the highest positive rate was reached after the seventh, eighth and ninth subcloning assays, but the positive rate decreased after the tenth subcloning. Combined with the fact that the two previous cell fusions with cloning had not been showing more than 90% inhibition of the subclonal plate, supernatants with different data were collected for competitive and non-competitive ELISA assays after the tenth subclonal assay. Cell supernatants were collected and tested using a non-competitive and indirectly competitive ELISA. The results are shown in Figure 5, where 1 and 2 are supernatants of cell wells with B0 > 0.6 and 1-B/B0 > 0.3 in the 96-well supernatant assay, and 3 and 4 are supernatants of cell wells with B0 > 1 and 1-B/B0 < 0.3 in the 96-well supernatant assay. The results showed that antibodies with high specificity and affinity for AFB_1_ were present in the supernatants of cell wells 1–4. The detection at a single standard concentration and without dilution of the supernatant may be due to the excessive number of antibodies in their supernatants. From the subclonal assay data, it was found that there is a disadvantage in choosing a single concentration standard to assay the cell supernatant. Selecting cells via direct assay without titration of the supernatant can mean some cells with good data are overlooked. Therefore, after the tenth, eleventh and twelfth subcloning, cell wells with B0 > 1.5 and in good condition were selected for screening cell lines using a three-step sample amplification and a multi-concentration standard assay.

#### 2.1.3. Selection of Positive Cell Lines and Purification of Antibodies

**Figure 6:**
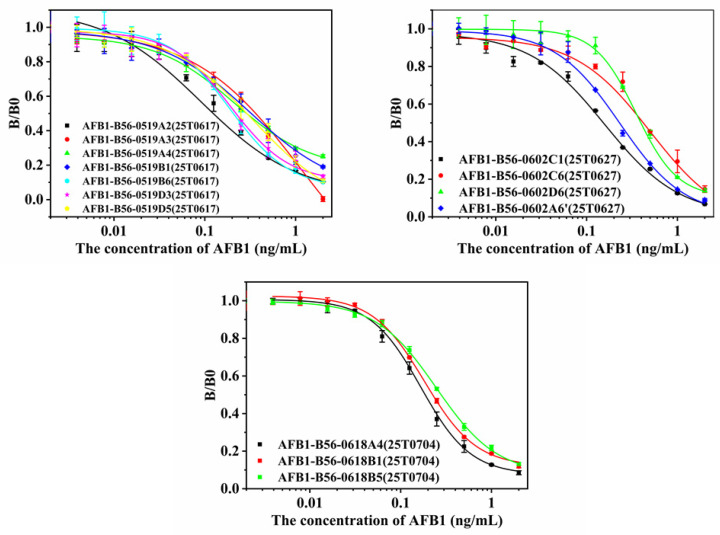
Competitive ELISA detection for AFB_1_ in 14 cell lines obtained by three-step amplification screening. The images were the test data of different batch expanded cells.

In the tenth to twelfth subclones of B56 mouse fusion cells 21, 30 and 20 cell lines were selected for expansion into 24-well cell plate culture, respectively. After two amplification and experimental screenings, four cell lines were identified. ELISA detection results of 14 cell lines are shown in Figure 6 and Table 1. All cell lines except AFB_1_-B56-0519D5 showed good sensitivity. Comparing the IC_50_ values and the magnitude of B0 values of the four cell lines, the final selection was AFB_1_-B56-0519A2 for antibody preparation. 

**Figure 7:**
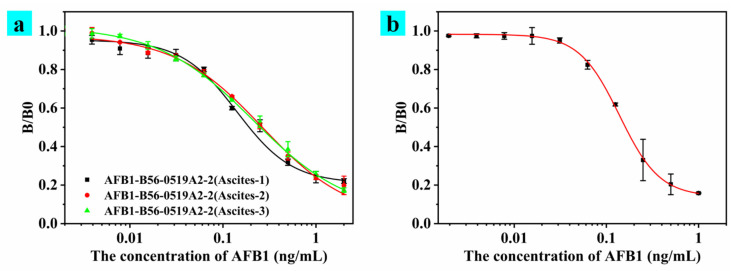
The results of antibody detection obtained via culture in mice. (**a**) Detection of antibodies in ascites from different mice. (**b**) Purified antibody competition assay data.

AFB_1_-B56-0519A2 cells were expanded and cultured, and cell suspension cells were injected into the abdominal cavity of mice previously injected with an incomplete Fredrick adjuvant to produce ascites purification antibodies. The test results of ascites of different mice and the antibody test results after purification using the ammonium capylate method are shown in Figure 7. The testing and recognition of ascites are shown in Figure 7a. The test results of different ascites consistently showed good stability. The purified antibody was named mAbA2-2, and the detection results are shown in Figure 7b. As shown in Table 2, the specific detection of mAbA2-2 showed that the antibody had good specificity against AFB_1_. An antibody typing kit was used to detect purified antibodies, which were mainly IgA, IgM and IgG1. The antibodies were analyzed via SDS-polyacrylamide gel electrophoresis, and two distinct bands were found.

### 2.2. Construction of MBs-icELISA

#### 2.2.1. Mechanism Explanation of MBs-icELISA

**Figure 8:**
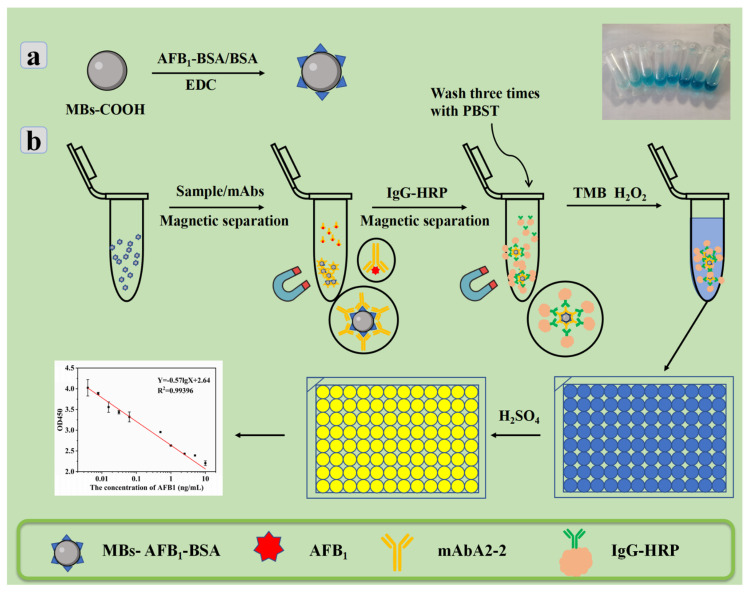
Schematic diagram of MBs-icELISA detection process based on dynamic pseudo-homogeneous magnetic bead competition. (**a**) MBs-AFB_1_-BSA preparation process. (**b**) The mechanism of MBs-AFB_1_-BSA detection of aflatoxins, using the different absorbance of the reaction solution at 450 nm for quantitative analysis.

While the conventional ELISA is implemented on an enzyme labeling plate, the MBs-icELISA changes the carrier to immunomagnetic beads, as shown in Figure 8. Figure 8a shows the fixation of antigens by magnetic beads via carboxyl groups. MBs-icELISA was accomplished with immunomagnetic beads as a carrier, combined with AFB_1_-BSA to form MBs-AFB_1_-BSA for subsequent capture (Figure 8b). The well-dispersed MBs-AFB_1_-BSA in MBs-ELISA acts as a stirrer to keep the solution in a homogeneous state both in the presence of a magnetic field and in the mixed state. The construction of MBs -icELISA was divided into four main parts. First, MBs-AFB_1_-BSA competes with AFB_1_ and targets mAb to form MBs-AFB_1_-BSA-mAbA2-2. Secondly, after magnetic separation, IgG-HRP was further identified with MBs-AFB_1_-BSA-mAbA2-2 to form a sandwich immune complex: MBs-AFB_1_-BSA-mAbA2-2-IgG-HRP. In the third step, the immune complex was separated and washed three times, and TMB color-developing substrate was added. After color development was completed using HRP, sulfuric acid was terminated, and finally a yellow reaction liquid was obtained. The absorbance of the reaction product of the terminated color-developing solution was determined at 450 nm and defined as OD450. 

#### 2.2.2. Optimization of MBs-AFB_1_-BSA Coupling

**Figure 9:**
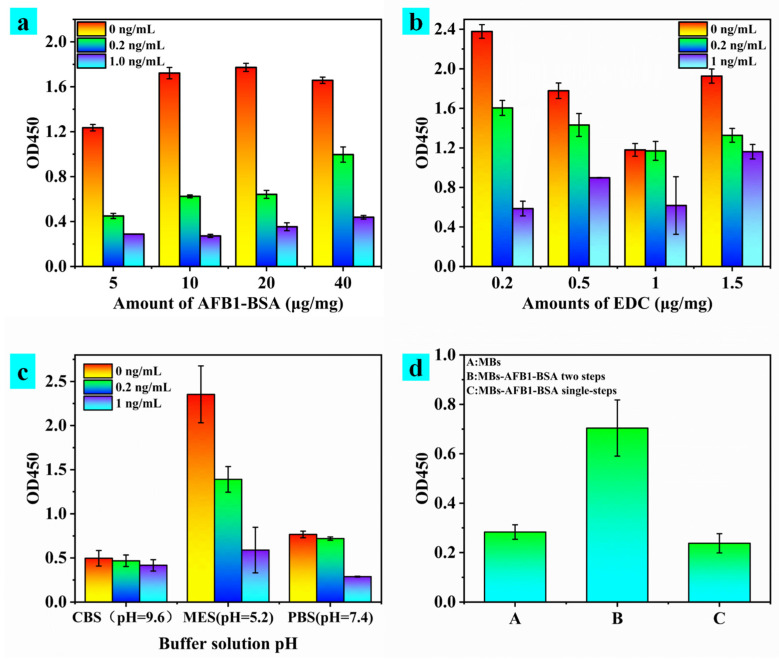
Absorbance at 450 nm of MBs-icELISA of MBs-AFB_1_-BSA obtained via different (**a**) concentrations of AFB_1_, (**b**) amounts of EDC, (**c**) pH and (**d**) steps.

MBs-AFB_1_-BSA, as a substrate, affected the sensitivity of the detection method from the beginning. AFB_1_-BSA dosage, 1-(3-Dimethylaminopropyl)-3-ethylcarbodiimide hydrochloride (EDC) dosage and pH of coupling buffer were selected for optimization, and the results are shown in Figure 9. The immunomagnetic beads obtained under different coupling conditions were added with 0, 0.2, and 1 ng/mL AFB_1_, respectively for detection via MBs-icELISA. Amounts of 0.2 and 1 ng/mL AFB_1_ were used to mimic low and high concentrations. The effect of coupling conditions on MBs-AFB_1_-BSA activity was evaluated by comparing OD450 and the inhibition rate. First, AFB_1_-BSA at 5–10 μg with the increase in concentration was also in the rising stage of OD450, and from 10–40 μg with the increase in concentration was at about the same OD450. Ten micrograms was selected as the amount of antigen coupling per mg of magnetic beads considering material loss (Figure 9a). Secondly, 0.2 μg EDC was suitable for the coupling of MBs and AFB_1_-BSA (Figure 9b). The function of EDC was to activate the reactive group. Insufficient EDC made the activation incomplete, and excessive EDC easily deactivated AFB_1_-BSA. Third, three buffer solutions with pH 9.6, 7.4, and 5.2 were selected to study the effect of the pH of the coupling buffer on OD450 values (Figure 9c), and the highest OD450 values and the highest inhibition were observed at pH 5.2 MES buffer solution. Finally, the capture process of antigen-antibody was optimized as shown in Figure 9d. Panel A was the case of only magnetic beads without adding antibody and secondary antibody, panel B was the case of adding antibody and enzyme-labelled secondary antibody in stages for incubation, and panel C was the case of adding antibody and enzyme-labelled secondary antibody together for incubation. Figure 9d shows that the combined effect of a phased addition was better than that of one-time addition.

#### 2.2.3. Optimization of MBs-icELISA Capture Process

**Figure 10:**
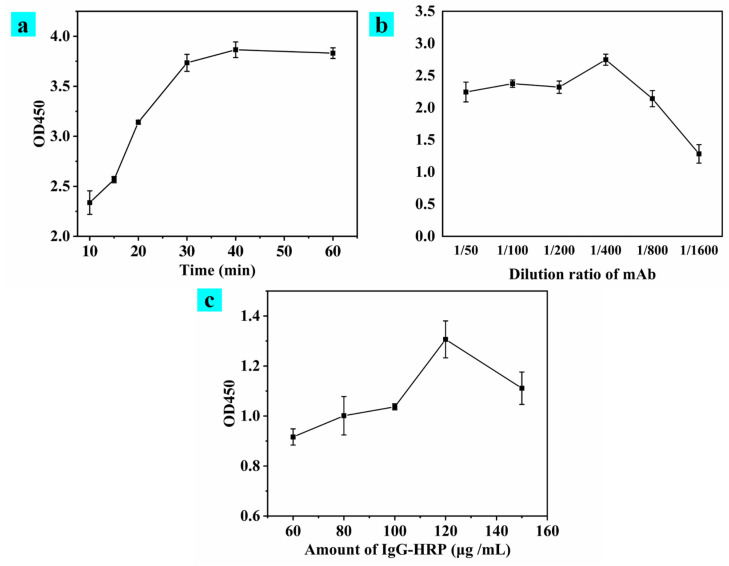
Absorbance at 450 nm of MBs-icELISA with (**a**) an incubation time of mAbA2-2, (**b**) a dilution ratio of monoclonal antibody, and (**c**) an amount of IgG-HRP.

In addition to the effect of the feeding amount during MBs-AFB_1_-BSA coupling, the numbers of reagents added during the reaction affected the sensitivity of MBs-icELISA. Therefore, the incubation time of mAbA2-2 and the amount of mAbA2-2 and IgG-HRP used were optimized, and the results of each optimization are shown in Figure 10. Firstly, the incubation time of mAbA2-2 increased gradually with the increase in the OD450 value over 5–30 min, and the OD450 value gradually stabilized after 30 min; the results are shown in Figure 10a. The incubation time of 30 min mAbA2-2 was selected. Secondly, mAb, as the most essential recognition element of MBs-icELISA, de-termined the sensitivity of the sensor; with the rise in mAb, OD450 gradually increased, reaching 1:400 after a stable trend. These results are shown in Figure 10b. Finally, the amount of IgG-HRP affected the colorimetric response of MBs-icELISA; too little IgG-HRP affected the sensor and the upper limit of detection, too much produced waste, and the amount of IgG-HRP was optimized with the purpose of conservation. The optimal amount of IgG-HRP was 120 μg/mL, as shown in Figure 10c.

#### 2.2.4. The Establishment of the MBs-icELISA Standard Curve

**Figure 11:**
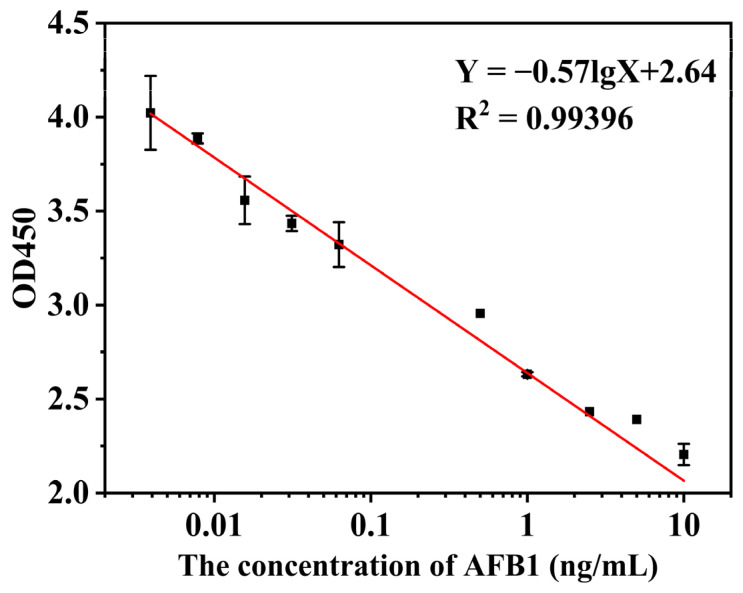
The linear relationship between AFB_1_ and absorbance in the detection of AFB_1_ by MBs-icELISA.

The sensitivity of MBs-icELISA to AFB_1_ was studied under optimal conditions. With the increase in the AFB_1_ concentration, the absorbance value of the sensor decreases gradually, as shown in Figure 11. MBs-icELISA had a good linear relationship in the concentration range of 0.004~10 ng/mL, and the linear range covers five orders of magnitude. The detection limit (LOD) for AFB_1_ was 0.0013 ng/mL. The results showed that the prepared monoclonal antibody had a good application prospect and showed good sensitivity and a wide recognition range when combined with MBs-icELISA.

## 3. Conclusions

In this study, we found that the traditional limited dilution subcloning method had some drawbacks in the preparation of aflatoxin monoclonal antibodies, and that single standard detection of antibodies would lead to some better cell lines being ignored. Therefore, the limited dilution subcloning method combined with three steps of sample amplification and multiple concentrations of standards was used in the cell-screening process to obtain aflatoxin monoclonal antibodies. MBs-icELISA was established using immunomagnetic beads as the carrier, and the dynamic homogeneous reaction process was achieved through the combination of MBs-AFB_1_-BSA and IgG-HRP. The addition of magnetic nanobeads facilitated the kinetics of the assay and enabled rapid separation and enrichment. Compared with traditional ELISA, the use of MBs-icELISA can greatly reduce detection time, improve sensitivity, simplify operations, and lower costs. MBs-icELISA also has a very good development prospect and is expected to be applied to the rapid detection of AFB_1_ in the field.

## 4. Materials and Methods

### 4.1. Materials and Apparatus

Aflatoxin B_1_-bovine albumin (AFB_1_-BSA), hypoxanthine-aminopterin-thymidine (HAT), hypoxanthine-thymidine (HT), polyethylene glycol 1450 (PEG 1450), 2-[4-(2-hydroxyethyl)piperazin-1-yl]ethanesulfonic acid (Hepes), L-glutamine (L-Gln), and Freund’s adjuvant were purchased from Sigma-Aldrich (St. Louis, MO, USA). RPMI-1640 medium was purchased from Cytiva (Beijing, China). Fetal bovine serum was purchased from Every Green (Zhejiang, China). Goat anti-mouse immunoglobulin G horseradish peroxidase (IgG-HRP) was purchased from Lvdu (Binzhou, China). Hybridoma Feeder adds factors were purchased from Boaolong (Beijing, China). Cell growth was observed using a BDS400 inverted microscope purchased from Chongqing AOte Optical Instrument, Chongqing, China. Absorbance was measured using a Varioskan LUX enzyme label purchased from Thermofisher (Waltham, MA, USA).

### 4.2. Animal Immunity and Cell Fusion

The SPF-grade female Balb/c mice (4–6 weeks) were ordered by Jinan Pengyue Experimental Animal Breeding Co., Ltd., Jinan, China. All animal experiments were carried out by with the ARRIVE guidelines and in accordance with the UK Animals (Scientific Procedures) Act, 1986 and associated guidelines, EU Directive 2010/63/EU for animal experiments, and the National Research Council’s Guide for the Care and Use of Laboratory Animals. Mouse sp2/0 myeloma cells were provided by Shandong Fenghua Biological Co., Ltd. (and cultured in Hyclone 1640-RPMI complete medium). Each mouse was immunized with 200 μL of the immunization reagent, which contained 50% volume of Freund’s adjuvant and 40 μg of AFB_1_-BSA. Immunization was performed via a subcutaneous multipoint injection on the back after complete emulsification of the immunizing reagent. Mice serum was collected by tail break and tested by ELISA. Well-immunized mice were selected for immunization with the same volume of immunogen shock three days before cell fusion. A sufficient number of myeloma cells cultured in advance were mixed with splenocytes in a 1:3 ratio in the presence of 50% PEG for cell fusion. The fused cells were cultured in 96-well cell plates with 100 mL of HAT medium.

### 4.3. Hybridoma Cell Supernatant Assay and Cellular Subcloning

The cell growth in the cell wells was observed at any time, and the cells were tested for antibody production when the cells in the cell wells had grown to a suitable state. Each cell well corresponds to two assays: one is a competitive ELISA with AFB_1_ added, and the other is a non-competitive ELISA without AFB_1_ added. The data obtained from the two assays correspond to the B and B0 values, respectively, and the 1-B/B0 value is the inhibition rate. The cell wells with good data and good cell status were selected for subcloning. For subcloning, the cells in the wells were counted and then diluted. One-hundred microliters of cell suspension with a concentration of 5 cells/mL was added to the first eight columns of the 96-well cell plate, and 100 μL of cell suspension with a concentration of 50 cells/mL was added to the last four columns.

### 4.4. Cellular Three-Step Sample Amplification

The cells in the 96-well cell plate were tested for antibodies and then expanded to the 24-well cell plate by selecting the appropriate cells according to the cell status. The cells in the 24-well plates were grown exponentially and the cell supernatants were collected for non-competitive and competitive indirect ELISA assays. Based on the assay data, cells with low IC_50_ values were selected and expanded into 6-well plates. After exponential cell growth, cell supernatants were collected again for non-competitive and competitive indirect ELISA assays. The cell lines were compared between the two assays, and cells with stable and low IC_50_ values were selected for expansion for antibody preparation.

### 4.5. Preparation and Identification of Antibodies

The hybridoma cells were diluted to 1–2 × 10^5^ with basal medium and injected into the peritoneal cavity of mice immunized intraperitoneally with Freund’s adjuvant 3–10 days in advance. The mice peritoneal fluid was collected when they were depressed, had prickly hair and were sluggish in movement. The collected ascites were centrifuged and the monoclonal antibody powder was prepared by ammonium octanoate method and freeze-dried. The prepared monoclonal antibodies were tested for specificity and affinity via an ELISA, and the antibody isotypes were detected using a Mouse Monoclonal Antibody Isotype Identification Test Kit purchased from Sigma-Aldrich (St. Louis, MO, USA).

### 4.6. AFB_1_-BSA Conjugated Beads (MBs-AFB_1_-BSA)

AFB_1_-BSA coupled magnetic beads were 500 nm carboxylated magnetic beads purchased from Bemag Biologicals, and the carboxyl group on the beads was used to biocouple with the amino group of AFB_1_-BSA. The magnetic beads containing 10 mg of the carboxyl group were first washed three times with MES buffer (0.05 M, pH 5.2). Then, 100 μg of ABF_1_-BAS monoclonal antibody (dialyzed with 0.05 M MES buffer) was added and the reaction was slowly shaken for 12 h. Subsequently, 0.5 mL of the special blocking agent was added and the reaction was maintained at room temperature for 4 h. The beads were washed three times with preservation buffer and stored at 4 °C.

### 4.7. Protocol of the MBs-icELISA

MBs-icELISA was performed as follows: 1 μL of MBs-AFB_1_-BSA prepared using the procedure detailed herein was washed three times with PBST, and 100 μL of different concentrations of AFB_1_ and 100 μL of mAbA2-2 at a dilution of 1:400 were added and mixed in 0.5 mL EP tubes in a mixer at 37 °C for 30 min. The tubes were then placed on a magnet for 5 s, and the supernatant was discarded. IgG-HRP was added in a mixer at 37 °C for 30 min. The immune complex (MBs-AFB_1_-BSA-mAbA2-2-IgG-HRP) was also added to the EP tubes. To the EP tube, goat anti-mouse IgG-HRP was added in a mixer at 37 °C for 30 min. The immune complex (MBs-AFB_1_-BSA-mAbA2-2-IgG-HRP) was washed 3 times with PBST in the presence of a magnet. TMB solution (200 μL) was added and incubated in the dark at 37 °C for 15 min. Briefly, 100 μL of the reaction solution was taken in a 96 micro-label plate, and 50 μL of 2 M sulfuric acid was added to terminate the reaction. The optical density (OD450) was measured using a Varioskan LUX multifunctional enzyme marker.

## Figures and Tables

**Table 1:** Summary of each index of culture bottle supernatant assay.

Cell Line	Titer	B0	IC_50_ (ng/mL)
AFB_1_-B56-0519A2	1/16	1.35930	0.14082
AFB_1_-B56-0519A3	1/32	1.28840	0.19655
AFB_1_-B56-0519A4	1/32	1.26475	0.49911
AFB_1_-B56-0519B1	1/24	1.20910	0.14153
AFB_1_-B56-0519B6	1/32	1.37770	0.29642
AFB_1_-B56-0519D3	1/32	1.39465	0.38429
AFB_1_-B56-0519D5	1/32	1.60165	47,895.84380
AFB_1_-B56-0602C1	1/128	0.72990	0.15767
AFB_1_-B56-0602C6	1/192	1.26010	0.50066
AFB_1_-B56-0602D6	1/ 64	1.37515	0.35058
AFB_1_-B56-0602A6′	1/512	1.03005	0.23179
AFB_1_-B56-0618A4	1/96	1.22790	0.16639
AFB_1_-B56-0618B1	1/512	1.08835	0.18250
AFB_1_-B56-0618B5	1/64	1.02876	0.24678

**Table 2:** Data on the specificity of mAbs for different toxins.

Toxin	IC_50_	CR%
AFB_1_	0.13522	100
AFB_2_	>2	<6.8
AFM_1_	>2	<6.8
AFM_2_	0.47521	28.5
AFG_1_	>2	<6.8
AFG_2_	1.0609	12.75
T-2	>2	<6.8
ZEN	>2	<6.81
DON	1.17463	11.52
OTA	>2	<6.8

## Data Availability

Date are contained within the article.
